# High-definition mapping of permanent junctional reciprocating tachycardia: Deeper insights into atrial activation and branching

**DOI:** 10.1016/j.hrcr.2026.01.024

**Published:** 2026-02-09

**Authors:** Philippe Maury, Amélie Clave, Maxime Beneyto, Deborah Foltran, Yves Dulac, Elena Kaafarani, Hubert Delasnerie, Clement Karsenty, Anne Rollin

**Affiliations:** 1 Cardiology Department, University Hospital Rangueil Toulouse, Toulouse, France; 2 I2MC, INSERM UMR 1297, Toulouse, France; 3 Boston Scientific, Voisins-le-Bretonneux, France; 4 Children Hospital, University Hospital Toulouse, Toulouse, France

**Keywords:** Permanent junctional reciprocating tachycardia, 3D mapping, Accessory pathway, Coronary sinus, Supraventricular tachycardia

**Key Teaching Points**

• Accessory pathway during permanent junctional reciprocating tachycardia is connected to the coronary sinus musculature.
• Atrial insertion of the accessory pathway (on the coronary sinus) may be larger than expected, explaining some issues with ablation.
• Earlier accessory potential may be recorded through a wide area.
• Proximal (ventricular) insertion of the accessory pathway could not be investigated.

## Introduction

Permanent junctional reciprocating tachycardia (PJRT), originally described by Coumel et al in 1967,^1^ is a very rare type of supraventricular tachycardia (1%), mostly seen in children and infants.^2^ PJRT involves an atypical accessory pathway (AP) with long and decremental unidirectional retrograde conduction,^3^ even if some more exceptional cases of anterograde conduction with overt preexcitation have been reported.^4^^,^^5^

Although many electrophysiological maneuvers have been developed for assessing the presence of such atypical AP, the precise substrate for the retrograde link of the reentry circuit is still poorly recognized. Bundles of the ordinary myocardium connecting the coronary sinus (CS) to the interventricular septum have been shown in a unique pathological investigation, without evidence of an accessory atrioventricular node and with fiber orientation changes and interstitial fibrosis.^6^^,^^7^ Such a tortuous and oblique path with fibrosis and fiber orientation changes was thought to be responsible for the decremental properties of conduction.

However, only the characteristics of the atrial insertion of the AP can be investigated in clinical practice. Even if AP potentials have sometimes been recorded,^3^^,^^5^^,^^8^, ^9^, ^10^ anatomical evaluation of the proximal part of the bypass tract is not feasible because of the usual lack of anterograde conduction and the impossibility to map the ventricular insertion of the AP.

PJRT can be successfully treated by targeting the atrial exit of the AP by ablation. Atrial exit is usually located at the right posteroseptal space, CS ostium, or proximal CS in most cases,^2^^,^^3^^,^^9^^,^^11^ although other various less common locations can be seen.^5^^,^^9^, ^10^, ^11^, ^12^, ^13^

Cases of 3-dimensional (3D) mapping of PJRT have been reported,^5^^,^^8^^,^^11^^,^^14^, ^15^, ^16^ demonstrating an atrial insertion of the AP in the proximal CS or CS ostium^8^^,^^14^^,^^15^ or the right inferior paraseptal region of the tricuspid annulus (cavo-tricuspid isthmus).^14^ However, these were not high-density mapping studies, including only a few electrograms at the site of earliest atrial activation; thus, detailed anatomical characteristics of the atrial insertion of the AP could not be investigated.

We present here a case of high-density mapping of retrograde atrial activation during PJRT, demonstrating a very large atrial insertion of the bypass tract, possibly branching on the CS musculature before activating the atrium.

## Case report

**Figure 1:**
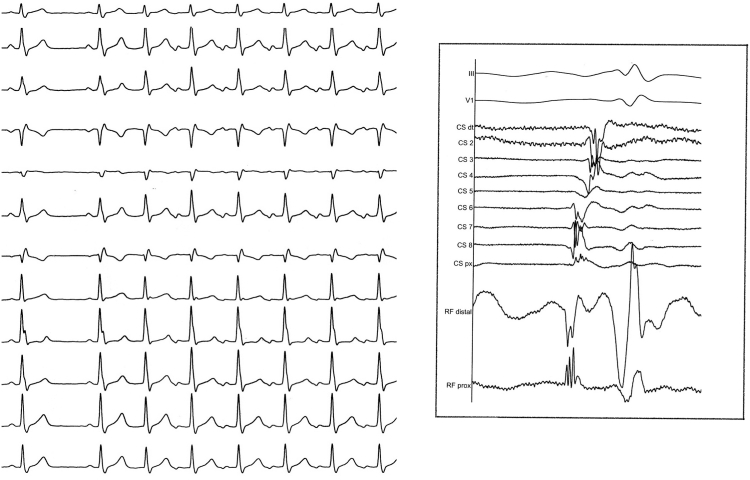
*Left:* 12-lead electrocardiogram of the tachycardia. *Right:* Intracardiac recordings at the site of earliest atrial activation during the tachycardia at the first procedure. CS = coronary sinus; RF distal and RF prox = distal and proximal dipoles of the radiofrequency ablation catheter.

A 12-year-old girl was referred for ablation of PJRT lasting for years, with ventricular rates previously controlled with β-blockers and digoxin. Tachycardia could be easily induced by atrial or ventricular extrastimuli or occurred spontaneously during stable sinus rhythm, with a long RP interval and biphasic P waves in the inferior leads (Figure 1). A His-refractory premature ventricular beat advanced the next atrial event with tachycardia resetting. Adenosine induced retrograde block and ceased the tachycardia, and a VAV pattern was observed after entrainment of the tachycardia by ventricular pacing.

During the first procedure, performed using fluoroscopy only, inefficient irrigated radiofrequency applications (25–30 W) were delivered at the earliest atrial activation (Figure 1) in the proximal CS, CS ostium, or lower septal tricuspid annulus, preceding left atrial activation, before mechanical bumping was observed in the middle part of the CS, where additional radiofrequency applications were delivered.

**Figure 2:**
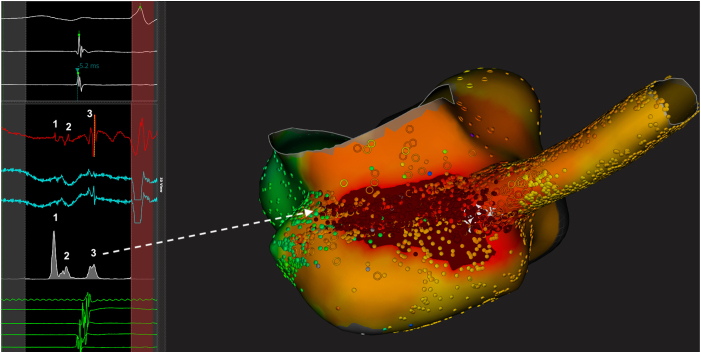
High-density 3-dimensional mapping of the atrial activation during PJRT, with depiction of the 3 diastolic potentials (sometimes recorded at the same area). **1** = area of a first potential possibly represented by the accessory pathway potential of the CS musculature (878 EGMs). **2** = area of a second potential probably represented by the CS musculature (6978 EGMs). **3** = remaining atrial activation (666 EGMs) (see text for explanation). *Left*: *White tracings*: Lead II and reference potential (CS). *Red and blue tracings*: Example of local bipolar (*red*) and unipolar (*blue*) EGMs from the corresponding area (*white arrow*). *Gray tracing*: “Trend” averaging the local EGMs from the same area. *Green tracings:* CS activation. *Right*: Inferior view of the right atrium and proximal coronary sinus. CS = coronary sinus; EGM = electrogram; PJRT = permanent junctional reciprocating tachycardia.

**Figure 3:**
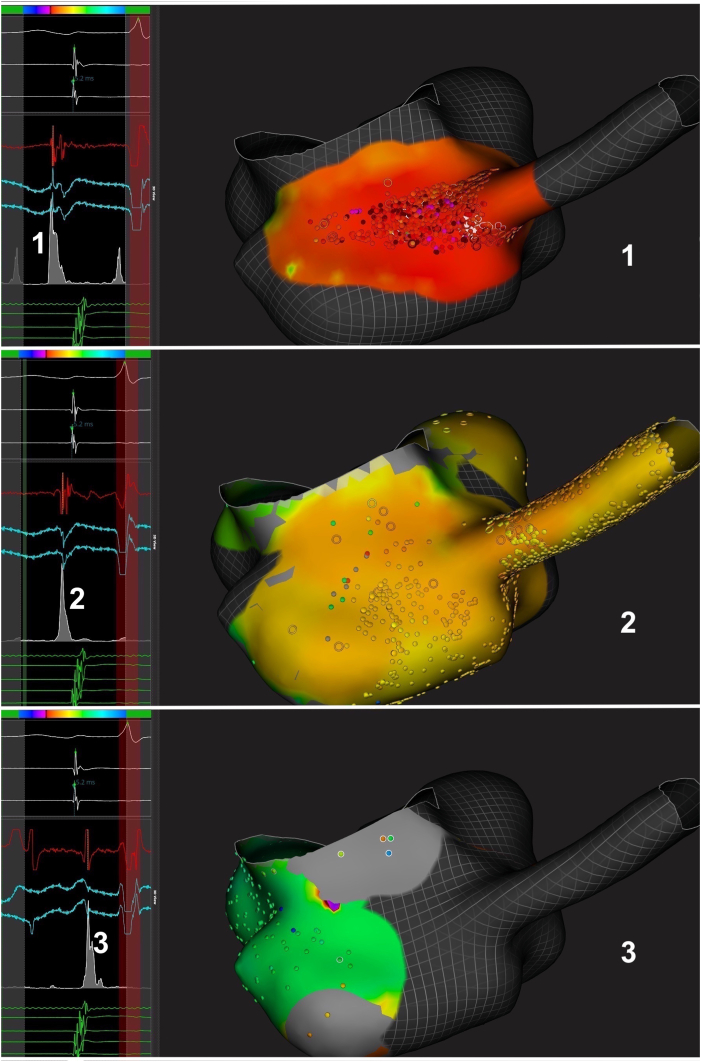
Sequential activation of the CS and neighboring areas during PJRT on the 3-dimensional map of the right atrium and CS (inferior view). **1** = area of a first potential possibly represented by the accessory pathway potential of the CS musculature (878 EGMs). **2** = area of a second potential probably represented by the CS musculature (6978 EGMs). **3** = remaining atrial activation (666 EGMs) (see text for explanation). *Left: White tracings:* Lead II and reference potential (CS). *Red and blue tracings:* Example of local bipolar (*red*) and unipolar (*blue*) EGMs from the corresponding area depicted on the right. *Gray tracing:* “Trend” averaging the local EGMs. *Green tracings:* CS activation. *Right*: Inferior view of the right atrium and proximal coronary sinus. Abbreviations as in Figure 2.

Unfortunately, the tachycardia recurred days afterward, and a new procedure was planned using high-density 3D mapping (Rhythmia, Boston Scientific, Inc.). Earliest diastolic fragmented potentials were present during PJRT at the CS ostium, covering a wide area extending below, above, and in front of the ostium (Figures 2 and 3), needing almost circumferential ablation, and needing multiple applications extending to the cavotricuspid isthmus and inferior vena cava, with several tachycardia interruptions before becoming noninducible. The patient presented with transient relapse some weeks after, and then permanent stable sinus rhythm was documented on repeated 24-hour recordings at 1 year.

Offline detailed analysis demonstrated 3 different areas of successive potentials in the area of atrial insertion (see Figures 2 and 3 and Supplemental Video 1): (1) a first roughly triangular area (first potential, with an R wave in the unipolar recording) at the junction between the inferior CS ostium and the inferior septal aspect of the cavo-tricuspid isthmus, covering 1.5 cm^2^ (20 × 7 mm) and depolarized in about 20 ms (activation velocity 75 cm^2^/s); (2) a second wider area centered by a second potential and extending from the remaining CS ostium and cavo-tricuspid isthmus up to the CS (15 cm^2^ in about 30 ms; activation velocity 500 cm^2^/s); and finally (3) the ascending activation of the remaining right atrium. Of note, the first 2 areas were in fact spatially merged (there were also electrograms with later potentials similar to area 2 in area 1). The delay between the local end of ventricular activation and the earliest atrial event was 225 ms, whereas the delay between the first and second diastolic signals was 45 ms and the delay between the second and the final atrial activation was 80 ms.

The first diastolic signal was interpreted either as a specific AP potential or as the first CS musculature activation, while the second activated area could have been the CS musculature (regardless of whether the first one was the AP potential or earlier CS activation with local block because of previous ablation).

## Discussion

This is the first reported case of PJRT evaluated using high-density mapping, demonstrating a large and complex pattern of retrograde atrial activation. Cases of 3D mapping of PJRT have been previously published,^5^^,^^8^^,^^14^, ^15^, ^16^ used in 32% of 172 ablation procedures in the largest multicenter registry of PJRT from 2014.^11^ However, high-density mapping with detailed activation sequences does not seem to have been reported to date.

Most APs involved in PJRT are branching at the CS ostium or above, below, and inside the CS,^9^ equivalent to the multiple sites of ablation in our case. Atrial insertion of the AP in PJRT may be more complex than imagined, with large CS muscular connections instead of fast conduction from a precise focal atrial breakthrough. This may explain some issues in ablation of PJRT.

The only case of a patient with PJRT who underwent pathological investigations showed tiny fibromuscular bundles crisscrossing the fat of the atrioventricular sulcus, connecting the lower rim of the CS outlet to the summit of the interventricular septum, with fiber orientation changes and interstitial fibrosis where they intermingle with the ventricular myocardium through narrow fissures of the atrioventricular annulus.^6^^,^^7^

Recording of 3 separate different potentials in the CS ostium and neighboring areas may be interpreted as specific AP potentials covering a relatively extended area, preceding a second, more wide area of CS musculature potentials, in turn preceding the final atrial activation. Specific AP potentials have been found in PJRT,^3^^,^^5^^,^^8^, ^9^, ^10^ although this could sometimes not be distinguished from fragmented atrial signals^9^ and not recorded earlier in the diastole,^9^ such as in our case. Presence of such potential at distant locations from the earliest atrial activation suggested a serpiginous course and more than one atrial connection.^3^ AP potential occurring very earlier during the diastole has also been reported, although more exceptionally and with very low-voltage signals,^3^^,^^9^ probably caused by a remote location of the AP and/or altered conduction. This was further validated in one case because of a changing timing of the potential depending on the existence of preexcitation.^9^ The CS musculature is known to connect to both the ventricle^17^ and the atrium,^18^ and the activation of the CS musculature can be recorded.^19^ We could not exclude that the first dual potentials rather represented fragmented CS musculature signals due to local block caused by the previous ablation lesions, but areas where second potentials were recorded exceeded the sites of previous ablation procedures in our opinion.

If our assumption is true, such atypical APs may have large muscular connections or may be thicker than usual Kent bundles with 1–3 mm diameter.^19^ This may explain the recurrences after the first procedure. However, it was described as “tiny atrial fibromuscular bundles” in the only available pathological description.^6^^,^^7^

The whole substrate between the end of ventricular activation at the basal ventricle and the first AP or CS activation could not be investigated here. We could only note that there was a 225-ms delay between the end of local ventricular activation and the very first diastolic signals but were unable to record any activation in this time window. Possibly, altered decremental conduction, more dependent on the calcium channel, may be more difficult to record using current systems, similar to atrioventricular node conduction.

## Disclosures

Ms Clave is an employee of Boston Scientific. The rest of the authors have no conflicts of interest.
