# Supplementary material for: High-definition mapping of permanent junctional reciprocating tachycardia: Deeper insights into atrial activation and branching
Source: HeartRhythm Case Rep. 2026 Feb 9;12(5):504–8. doi: 10.1016/j.hrcr.2026.01.024 (PMC13198266; doi:10.1016/j.hrcr.2026.01.024)
Supplement: Supplementary Material [file mmc2.doc]

Legend to movie: inferior view of the right atrium and coronary sinus showing the propagation of the successive waves of activation during tachycardia as depicted in fig 2 and 3
